# A nitronyl nitroxide and its two 1D chain Cu–Tb complexes: synthesis, structures, and magnetic properties†

**DOI:** 10.1039/d0ra00018c

**Published:** 2020-02-27

**Authors:** Meng Yang, Xiaohong Liang, Yandie Zhang, Zhijian Ouyang, Wen Dong

**Affiliations:** Guangzhou Key Laboratory for Environmentally Functional Materials and Technology, School of Chemistry and Chemical Engineering, Guangzhou University, Guangzhou Higher Education Mega Center 230 Wai Huan Xi Road Guangzhou 510006 P. R. China yangmeng@gzhu.edu.cn dw320@aliyun.com

## Abstract

A new nitronyl nitroxide, namely NIT-Ph-*p*-OCH_2_trz (1), and two types of one-chain Cu–Tb complexes, namely [TbCu(hfac)_5_NIT-Ph-*p*-OCH_2_trz·0.5C_6_H_14_]_*n*_ (2a) and [LnCu(hfac)_5_NIT-Ph-*p*-OCH_2_trz]_*n*_ (2b) (NIT-Ph-*p*-OCH_2_trz = 2-(4-((1*H*-1,2,4-triazol-1-yl)methoxy)phenyl)-4,4,5,5-tetramethylimidazoline-1-oxyl-3-oxide; hfac = hexafluoroacetylacetonate), have been successfully synthesized simultaneously through reacting nitronyl nitroxide radical NIT-Ph-*p*-OCH_2_trz with Cu(hfac)_2_ and Ln(hfac)_3_, and the molecular structures have been elucidated *via* single-crystal X-ray structural analysis. 2a features a ‘ladder-like’ chain structure, while 2b displays a linear chain structure built up by Ln(hfac)_3_ units bridged with the NO groups of radical ligands. The additional Cu(ii) ions are coordinated to the nitrogen atoms of the triazole units. Nonzero out-of-phase signals are observed for the Tb derivatives (2a and 2b) and they exhibit frequency-dependent out-of-phase signals indicating a single-chain magnet behavior.

## Abstract 2

A new nitronyl nitroxide and two types of one-chain Cu–Tb complexes have been successfully synthesized, and the molecular structures have been elucidated *via* single-crystal X-ray structural analysis.

## Graphical abstract

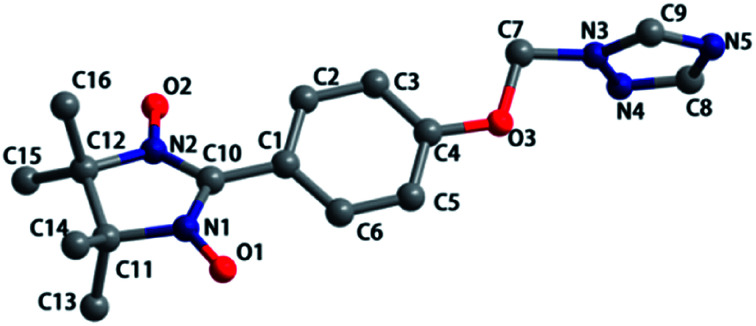

## Introduction

**Scheme 1:**
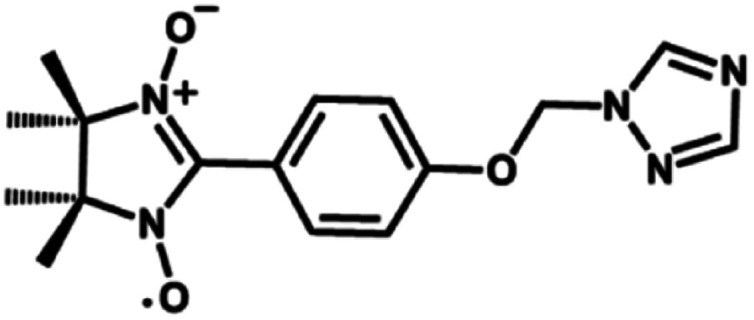
NIT-Ph-*p*-OCH_2_trz radical ligand.

Paramagnetic nitronyl nitroxide radicals (NITRs) are promising building blocks for the synthesis of novel heterospin molecular magnetic materials.^1,2^ First, the NO groups of the nitronyl nitroxides coordinate directly to metal ions, resulting in a strong magnetic coupling that reduces the quantum tunneling of the magnetization (QTM).^3–5^ Second, nitronyl nitroxide radicals are stable in air and are easily modified with functional groups to enhance the chemical properties of the material. A number of metal complexes based on nitronyl nitroxides, including 2p–3d, 2p–4f heterobispin and 2p–3d–4f heterotrispin complexes, have been synthesized in recent years.^6–8^ Moreover, metal-radical complexes are good derivatives for studying magneto-structural correlation, which not only is important to understand the magnetic exchange interactions between the metal and the radical, but also for the design of novel nitronyl nitroxide radical ligands for the development of new molecular magnetic materials. To design more complexes with topological structures, special attention has been paid to functionalized radicals. Nitronyl nitroxide radicals that contain a triazole ring are good derivatives because these free radicals can bond to the metal ions *via* the triazole nitrogen atoms and the radical oxygen atoms, allowing for the formation of polynuclear clusters.^9,10^ Herein, a functional NITR containing triazole ring named NIT-Ph-*p*-OCH_2_trz (NIT-Ph-*p*-OCH_2_trz = 2-(4-((1*H*-1,2,4-triazol-1-yl)methoxy)phenyl)-4,4,5,5-tetramethylimidazoline-1-oxyl-3-oxide, Scheme 1) was synthesized. Using this radical, two types of one-chain 2p–3d–4f complexes, namely [TbCu(hfac)_5_NIT-Ph-*p*-OCH_2_trz·0.5C_6_H_14_]_*n*_ and [LnCu(hfac)_5_NIT-Ph-*p*-OCH_2_trz]_*n*_, were obtained. Complex 2a exhibits a ladder-type chain structure, while complex 2b features a 1D Ln chain bridged by the NO moieties of the radical ligands. Both 2a and 2b show frequency-dependent out-of-phase signals in a zero field with an oscillation of 3 Oe, indicating single-chain magnet behaviors.

## Experimental section

### Materials and physical measurements

All reagents were purchased from commercial sources and used without further purification. Elemental analysis for C, H, and N was performed using a PerkinElmer elemental analyzer model 240. Powder X-ray diffraction (PXRD) data for all seven complexes were collected at room temperature on a Rigaku Ultima IV diffractometer using graphite monochromated Cu-Kα radiation. Magnetic measurements were measured on a SQUID VSM and PPMS-9 magnetometer. The magnetic susceptibilities were corrected for the diamagnetic contribution of the constituent atoms using Pascal's constants.

### Synthesis

#### Preparation of compound 1

The radical ligand of NIT-Ph-*p*-OCH_2_trz was synthesized according to literature methods.^11^ Yield: 0.61 g (52%). Anal. calcd for C_16_H_20_N_5_O_3_(%): C, 58.17; H, 6.10; N, 21.20. Found: C, 58.24; H, 6.12; N, 21.26.

#### Preparation of complexes of 2a and 2b

Tb(hfac)_3_·2H_2_O (0.0150 g, 0.02 mmol) was dissolved in 20 mL of dry boiling hexane and the solution was heated to reflux for 4 hours. Then, a solution of NIT-Ph-*p*-OCH_2_trz (0.0064 g, 0.02 mmol) in 3 mL of dry CHCl_3_ was added. The solution was refluxed for 30 minutes, and then solid Cu(hfac)_2_ (0.0098 g, 0.02 mmol) was added. The resulting solution was stirred for 20 minutes and then cooled to room temperature and filtered. The filtrate was kept at room temperature; after two days, blue needle-like crystals (2a, yield: 30%) and green block crystals (2b, yield: 28%), suitable for X-ray diffraction were obtained using the evaporation method. Anal. calcd for C_44_H_32_CuF_30_N_5_O_13_Tb (2a) (%): C, 32.40; H, 1.98; N, 4.29. Found: C, 32.36; H, 1.95; N, 4.26. IR(KBr): 1651(s), 1501(m), 1474(m), 1256(s), 1226(s), 1142(s), 801(m), 662(m), 587(m) cm^−1^. Anal. calcd for C_41_H_25_CuF_30_N_5_O_13_Tb (2b) (%): C, 31.01; H, 1.59; N, 4.41. Found: C, 31.05; H, 1.61; N, 4.39. IR(KBr): 3247(s), 1706(s), 1610(s), 1482(m), 1261(s), 1222(s), 1163(s), 1007(s), 767(s), 727(s) cm^−1^.

#### X-ray structure determination

All the crystal structures were determined using a Rigaku Saturn CCD diffractometer employing graphite-monochromated Mo-Kα radiation at 113 K. The structures of the ligands and Tb complexes were solved by direct methods using the SHELXS-2014 program and refined by full-matrix least-squares on *F*^2^ with SHELXL-2014.^12^ All the non-hydrogen atoms were refined anisotropically and the hydrogen atoms were added geometrically and refined as riding atoms with a uniform value of *U*_iso_. CCDC: 1973721 for 1, 1973720 for 2a and 1973722 for 2b.

## Results and discussion

### Crystal structures

**Fig. 1:**
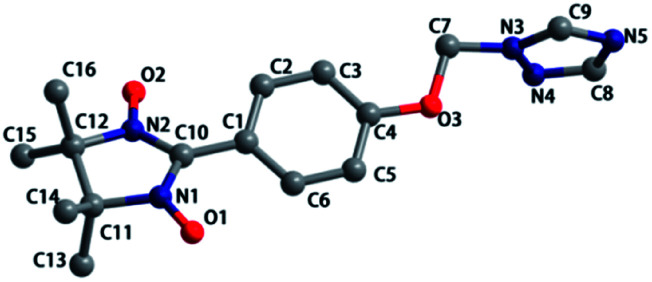
Molecular structure of 1. Hydrogen atoms are not shown for the sake of clarity.

Compound 1 crystallized in the orthorhombic space group *Pbca*. The molecular structure is shown in Fig. 1, and the summary of the detailed crystallographic data and structure refinement are given in Table 1. Selected bond lengths and angles are given in Table S1.† The bond lengths of O–N are 1.275(3) and 1.290(2) Å, and the corresponding N–O distances in the nitroxides range from 1.25 to 1.32 Å. The dihedral angle between the benzene ring and the NO–C–NO group is 21.786(72)°, while the dihedral angle between the benzene ring and the triazole ring is 88.420(67)°. As shown in Fig. S1,† a 1-D chain structure with ‘head-to-head’ and ‘tail-to-tail’ is formed by hydrogen bonding, and the distances of C⋯O are 3.566 and 3.662 Å. The compound packing along the *b*-axis displays a wavelike form.

**Table 1:** Crystallographic data and structure refinement summary for 1 and 2

Complex	1	2a	2b
Formula	C_16_H_20_N_5_O_3_	C_44_H_32_CuF_30_N_5_O_13_Tb	C_123_H_72_Cu_3_F_90_N_15_O_39_Tb_3_
Formula weight	330.37	1631.23	4761.40
*T* (K)	113(2)	113(2)	113(2)
Crystal system	Orthorhombic	Triclinic	Monoclinic
Space group	*Pbca*	*P*1̄	*P*2_1_/*c*
*a* (Å)	9.2257(18)	12.654(3)	18.785(3)
*b* (Å)	16.713(3)	13.533(3)	17.138(3)
*c* (Å)	21.020(6)	18.382(4)	52.309(10)
*α* (deg)	90	83.29(3)	90
*β* (deg)	90	87.52(3)	96.852(4)
*γ* (deg)	90	71.40(3)	90
*θ/*(deg)	1.94–27.92	1.60–27.99	3.0–27.5
*V* (Å^3^)	3241(11)	2962.9(13)	16 720(5)
*Z*	8	2	4
*D*_c_ (g cm^−3^)	1.354	1.828	1.891
*μ* (mm^−1^)	0.097	1.699	1.804
Unique reflns, *R*_int_	3880	10 301	36 573
	0.0789	0.0318	0.1375
GOF	1.010	1.002	1.053
*R*_1_	0.0696	0.0508	0.0686
w*R*_2_ (*I* > 2*σ*(*I*))	0.1615	0.1360	0.1577
*R*_1_	0.1059	0.0595	0.1084
w*R*_2_ (all data)	0.1875	0.1460	0.1857

**Fig. 2:**
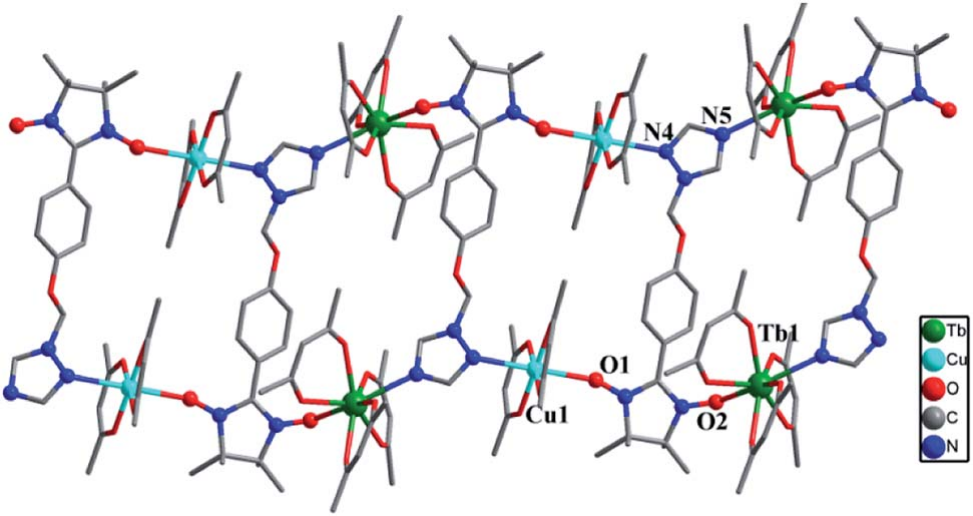
Molecular structure of complex 2a (hydrogen and fluorine atoms are not shown for the sake of clarity).

**Fig. 3:**
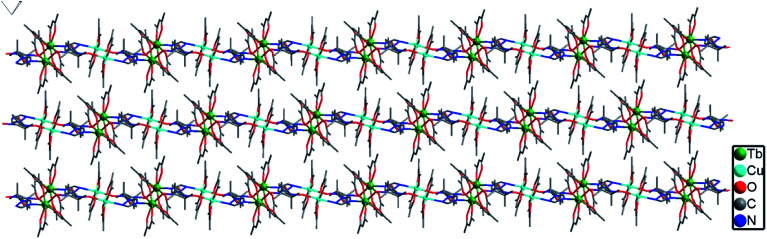
Crystal packing of complex 2a (all hydrogen and fluorine atoms are omitted for clarity).

Complex 2a crystallized in the triclinic *P*1̄ group space. As shown in Fig. 2, each Tb(iii) ion is coordinated to one N atom from the triazole ring of the ligand, six O atoms from the three hfac ligands and one O atom from the NO group of the nitronyl nitroxide radical, resulting in a distorted square-antiprismatic geometry (*D*_4d_; Table S3†). The data was analyzed using the SHAPE software.^13^ The Tb–O(radical) and Tb–N distances are 2.346(5) and 2.578(6) Å, respectively, while the Tb–O(hfac) distances range from 2.327(4) to 2.363(3) Å. The Cu(ii) ion is in a {NO_5_} coordinate environment in which the axial positions are occupied by one O atom (O1) from the NO group and one N atom from the triazole ring of another molecule. The Cu–O and Cu–N bond distances are 2.408(5) and 2.467(6) Å in the axis positions, while the Cu–O bond lengths range from 1.941(6) to 1.953(5) Å. The paramagnetic organic ligand is linked to two Tb(iii) and two Cu(ii) ions *via* its two NO groups and two N atoms of the triazole ring in a tetradentate μ_4_-η^1^:η^1^:η^1^:η^1^ mode, resulting in a ladder-like arrangement in which the anisole moieties form the rungs of the ladder. The Tb⋯Cu separations through the NIT motif and triazole ring are 8.50 and 6.93 Å, respectively. The Tb⋯Tb and Cu⋯Cu distances across the rungs are 8.67 and 11.09 Å, respectively (Fig. 3).

**Fig. 4:**
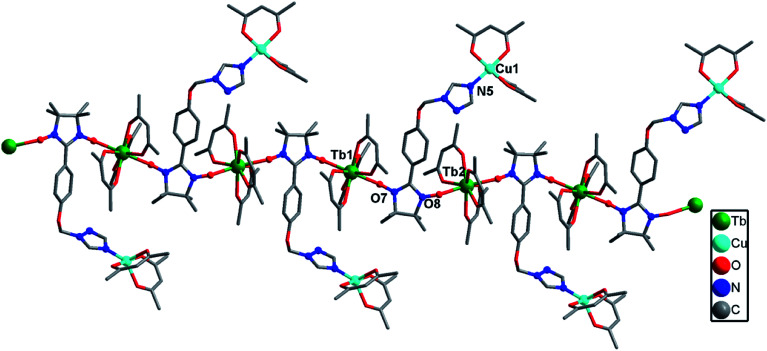
Molecular structure of complex 2b (H and F atoms are not shown for the sake of clarity).

**Fig. 5:**
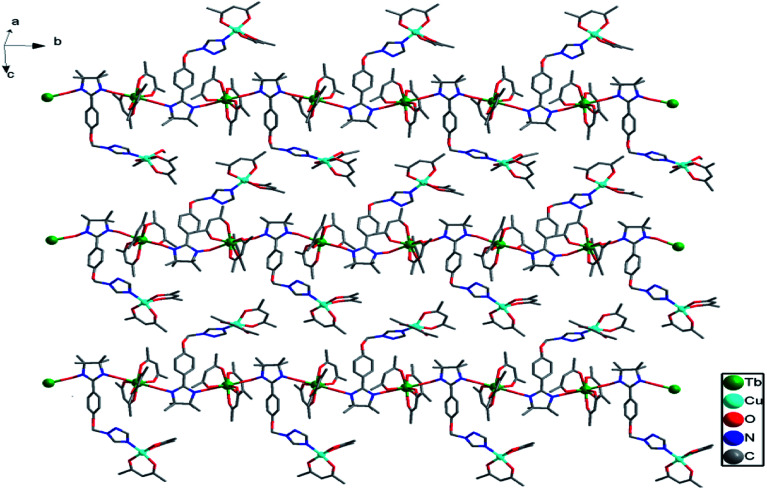
Crystal packing of complex 2b (all hydrogen and fluorine atoms are omitted for clarity).

Complex 2b was solved in the monoclinic system with the space group *P*2_1_/*c* and the asymmetric unit contains one [TbCu(hfac)_5_(NIT-Ph-*p*-OCH_2_trz)]_2_ and a [TbCu(hfac)_5_ (NIT-Ph-*p*-OCH_2_trz)] unit, which results in two crystallographically independent 1D chains in the crystal lattice. Compared to complex 2a, the linear chain in 2b is constructed from the radical ligand and Tb(iii) spin carriers, and the Cu(ii) ions are located on the side-chain. In complex 2b, each radical behaves as a tridentate ligand and binds two ions and one Cu(ii) ion in the μ_3_-η^1^:η^1^:η^1^ mode. The NO groups of the NIT-Ph-*p*-OCH_2_trz radical bridges two Tb(hfac)_3_ units to construct a 1D chain. Each Tb(iii) ion is coordinated to two O atoms from two radical ligands and six O atoms from three hfac ligands (Fig. 4). Shape analysis indicated that all of the Tb(iii) ions are located in distorted square-antiprismatic geometries (*D*_4d_; Table S3†). The Tb–O(hfac) bond lengths are in the same range as those of 2b (Table S5†). The Cu(ii) ions in 2b are all five-coordinated (Fig. S3†). The Cu1 and Cu3 atoms are both in the distorted square-pyramidal geometry (Table S4†). The equatorial planes are occupied by three O atoms from hfac ligands and one N atom from the triazole unit, while one O atom from the hfac ligand occupies the apical site. The Cu–N bond distances are 1.979(7) Å for Cu1 and 1.968(7) Å for Cu3. The Cu–O bonds in the equatorial plane range from 1.911(6) to 1.957(6) Å for Cu1 and Cu3, while the apical Cu–O bond distances are 2.158(9) and 2.167(10) Å for Cu1 and Cu3, respectively. The Cu2 atom is located in a trigonal bipyramidal environment (Table S4†). Its equatorial plane is formed by two O atoms (O23 and O25) from two hfac ligands and one N atom (N10) from the triazole unit; the apical positions are occupied by two hfac O atoms (O24 and O26). The axial Cu–O distances are 2.158(9) and 1.908(7) Å. The packing of the chains in complex 2b is shown in Fig. 5. The shortest intrachain Tb⋯Tb distance bridged by two NO groups is 8.57 Å, while the nearest interchain Tb⋯Tb, Cu⋯Tb, and Cu⋯Cu separations were found to be 10.05, 9.24, and 9.34 Å, respectively.

### Magnetic properties

**Fig. 6:**
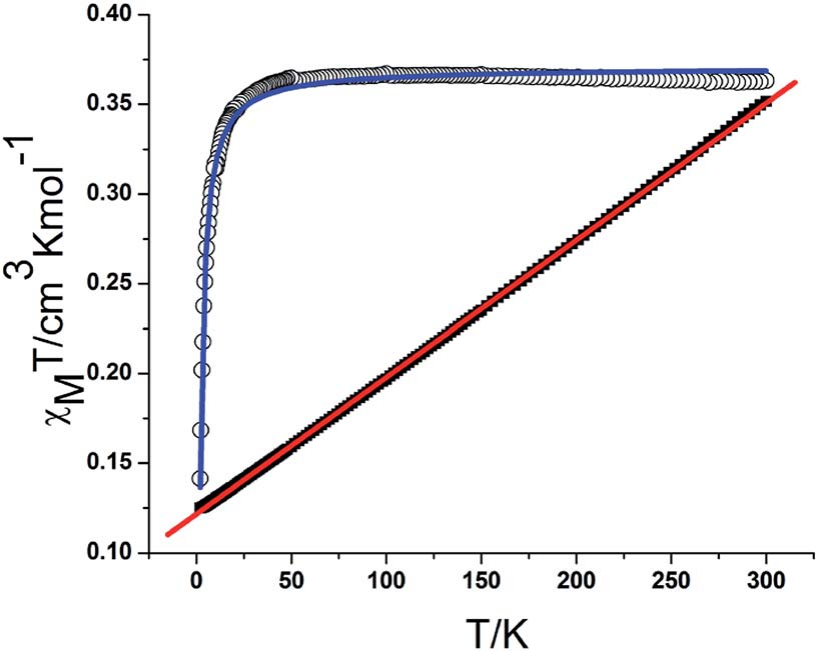
Plot of *χ*_m_*T* and *χ*_m_^−1^*versus T* for 1. The blue and red solid lines represent the fit of the experimental data to eqn (1) in the text and the Curie–Weiss law, respectively.

The magnetic susceptibilities of complexes 2 were measured on polycrystalline samples. The phase purity of the samples was verified by comparing experimental and simulated PXRD pattern XRD analyses (Fig. S4†). The variable temperature magnetic susceptibility data for compounds 1 and 2 were measured in the 2–300 K range. As shown in Fig. 6, the *χ*_m_*T* product of 1 at 300 K is 0.366 cm^3^ K mol^−1^, which is a little lower than the theoretical value of 0.375 cm^3^ K mol^−1^ for an isolated *S* = 1/2. With decreasing temperature, the *χ*_m_*T* value gradually decreases to a minimum value of 0.141 cm^3^ K mol^−1^ at 2 K. The 1*/χ*_m_*versus T* curve in the 2–300 K range follows the Curie–Weiss law with a Curie constant of *C* = 0.365 cm^3^ K mol^−1^ and a Weiss constant of *θ* = −0.72 K, which indicate that there is weak antiferromagnetic coupling between the NIT-Ph-*p*-OCH_2_trz radicals arising from two types of hydrogen bonding (3.566 and 3.6628 Å) between the radical ligands. According to the structure, the magnetic behavior of compound 1 can be treated as alternating 1D chains with *S* = 1/2. The magnetic data was analyzed using a theoretical expression^1^ deduced from the spin Hamiltonian *Ĥ* = −[*J*(*Ŝ*_1_ + *Ŝ*_2_) + *αJ*(*Ŝ*_2_ + *Ŝ*_3_)]. The experimental data was a good fit with *J* = −2.71 cm^−1^, *g* = 1.99, and *α* = 0.36. The negative value of *J* confirms the antiferromagnetic activity of the ligands.1
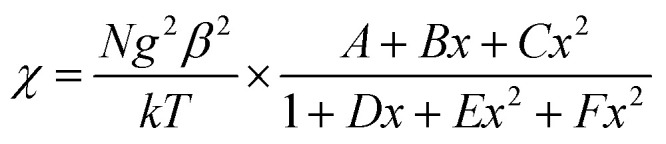
*χ* = |*J*|/*kT*; 0 ≤ *α* ≤ 0.4; *A* = 0.25; *B* = −0.062935 + 0.11376*α*; *C* = 0.0047778 − 0.033268*α* + 0.12742*α*^2^ − 0.32918*α*^3^ + 0.25203*α*^4^; *D* = 0.053860 + 0.70960*α*; *E* = 0.00071302 − 0.10587*α* + 0.54883*α*^2^ − 0.20603*α*^3^; *F* = 0.047193 − 0.0083778*α* + 0.87256*α*^2^ − 2.7098*α*^3^ + 1.9798*α*^4^

**Fig. 7:**
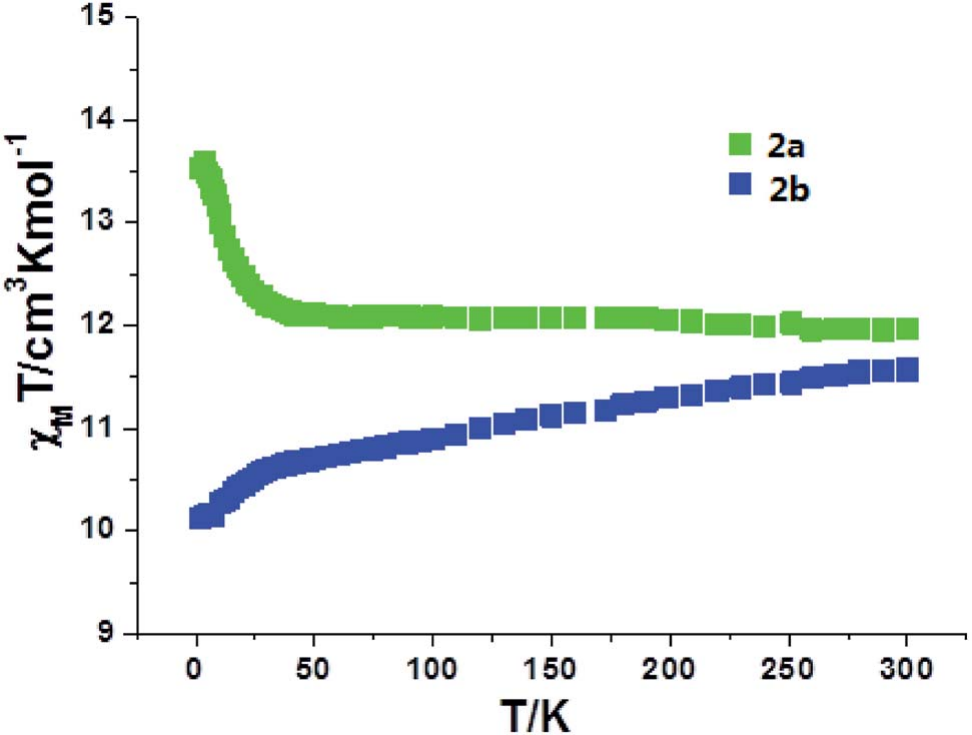
Plot of *χ*_m_*T versus T* for complexes 2a and 2b.

**Fig. 8:**
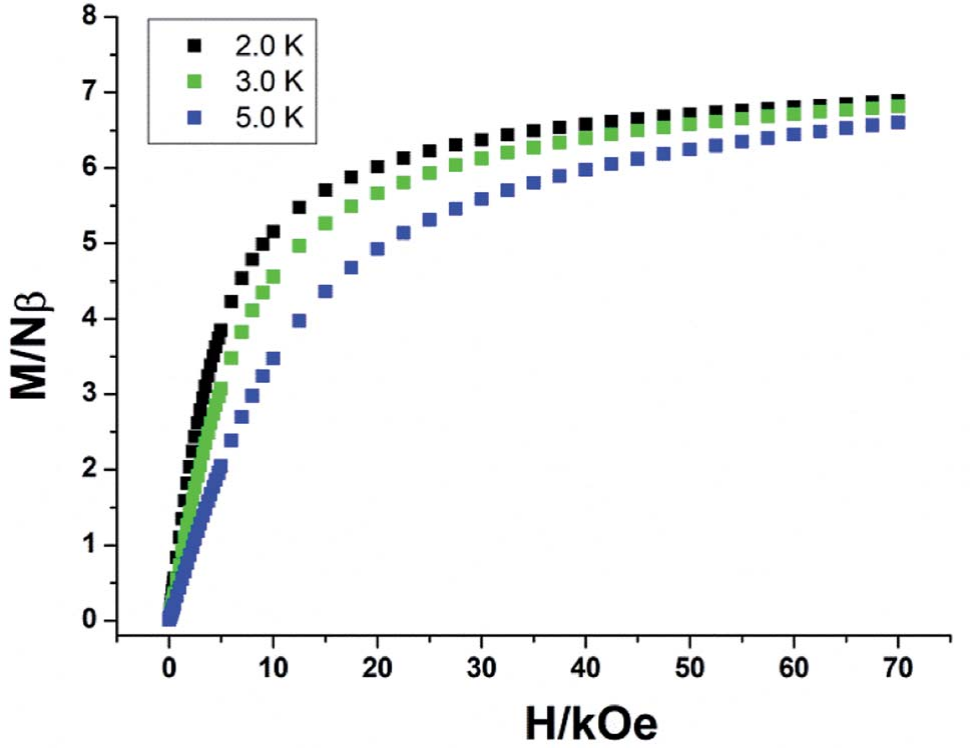
*M versus H* plot at 2, 3, and 5 K for complex 2a.

For complexes 2a and 2b, as shown in Fig. 7, the *χ*_m_*T* values are 12.10 and 11.58 cm^3^ K mol^−1^ at room temperature, slightly lower than the expected value of 12.57 cm^3^ K mol^−1^ for one uncorrelated Tb(iii) ion (^7^F_6_, *S* = 3, *L* = 3, and *g* = 3/2) plus one isolated Cu(ii) ion (*S* = 1/2, *g* = 2.0) and one radical (*S* = 1/2, *g* = 2.0). For 2a, the *χ*_m_*T* value is constant from 300 K to 25 K, then the value sharply increases to a maximum of 13.60 cm^3^ K mol^−1^ at 4 K. The value decreases to 13.52 cm^3^ K mol^−1^ at 2 K. The increase in the *χ*_m_*T* value at low temperatures confirms the ferromagnetic interactions between the Tb(iii) ions and the coordinated NO groups of the organic radicals, while the decrease in the *χ*_m_*T* value is attributed to the crystal field effects of the Tb(iii) ion. For 2b, the value of *χ*_m_*T* decreases slowly to a value of 10.52 cm^3^ K mol^−1^ at 25 K, and then steeply decreases to 9.94 cm^3^ K mol^−1^ at 2 K. This can be ascribed to the stronger crystal field effect of the Tb ion, which overwhelms the ferromagnetic radical-Tb contributions on the *χ*_m_*T* curve.^14^ Both complexes 2a and 2b show linear regions ranging from 9 K to 26 K and 4 K to 9 K, and the values of Δ*ξ* are 1.04 K and 0.22 K, confirming the 1D Ising-like character of these complexes (Fig. S5†). The *M versus H* curves of 2a and 2b at 2.0, 3.0, and 5.0 K show that the values of the magnetization increase with the applied dc field and do not reach saturation (Fig. 8 and S6†). The observation suggests that there exists significant magnetic anisotropy in 2a and 2b.

**Fig. 9:**
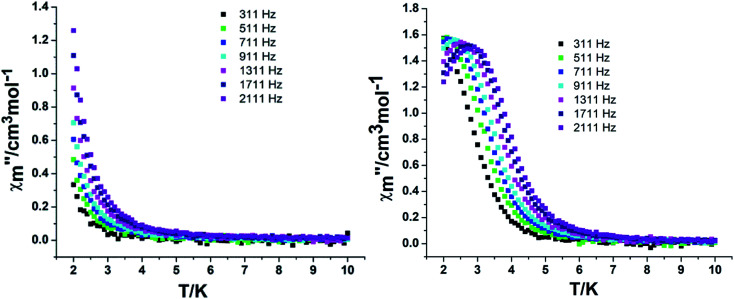
Temperature dependence of the out-phase in a zero (left) and 2 kOe field (right) for complex 2a.

**Fig. 10:**
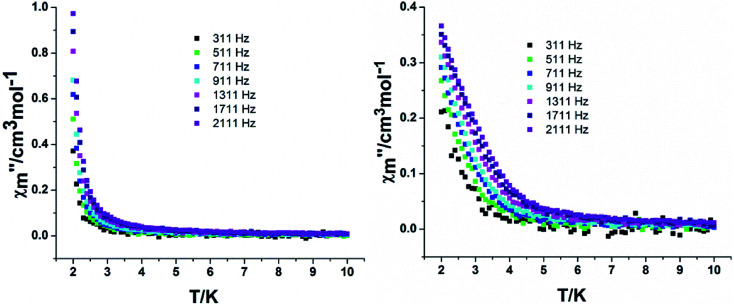
Temperature dependence of the out-phase in a zero (left) and 2 kOe field (right) for complex 2b.

In order to assess the relaxation dynamics of 2a and 2b, ac magnetic susceptibility measurements were performed with and without a static magnetic field. For complexes 2a and 2b, the frequency dependence of the out-of phase 
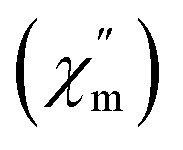
 signals are observed under zero dc fields, revealing slow relaxation of its magnetization (Fig. 9 and 10). However, no peak maxima are observed for 
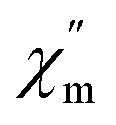
 above 2 K. The anisotropic energy barrier and *τ*_0_ values can be obtained from the fits of the ac susceptibility data using the equation ln(*χ*′′/χ′) = ln(*ωτ*_0_) + Δ_eff_/*k*_B_*T*.^15^ The best fit produced *τ*_0_ ≈ 1.90 × 10^−5^ s and Δ_eff_/*k*_B_ ≈ 5.93 K for 2a and *τ*_0_ ≈ 6.38 × 10^−6^ s and Δ_eff_/*k*_B_ ≈ 3.84 K for 2b (Fig. S9†). A static magnetic field of 2 kOe was applied to suppress the quantum tunneling process and the in-phase and out-phase susceptibility curves displayed obvious peaks for 2a. Unfortunately, there was no peak for complex 2b.

**Fig. 11:**
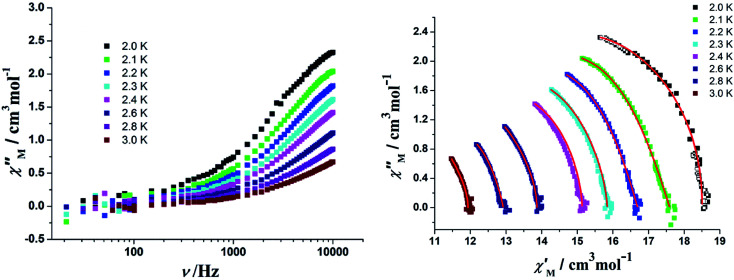
(Left) Frequency dependence of the out-of-phase components for the ac magnetic susceptibility in a zero field for 2a. (Right) Cole–Cole plots for 2a. The solid lines represent the best fits with modified Debye functions (see the text).

**Fig. 12:**
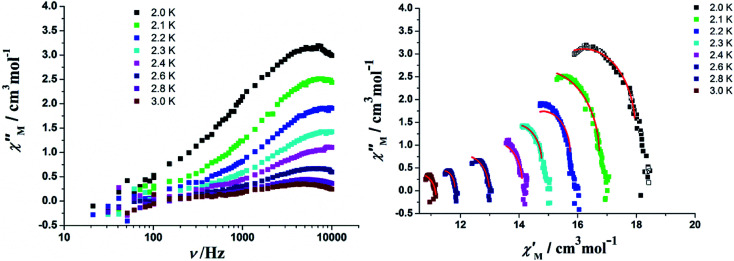
(Left) Frequency dependence of the out-of-phase components for the ac magnetic susceptibility in a zero field for 2b. (Right) Cole–Cole plots for 2b. The solid lines represent the best fits with modified Debye functions (see the text).

In order to perform an in-depth study on the relaxation dynamics of 2a and 2b, the frequency dependence of the *χ*′ and *χ*′′ signals were also examined in a frequency range of 10–10 000 Hz without applying a dc field. For complexes 2a and 2b, both the *χ*′ and *χ*′′ components of the alternating-current (ac) susceptibility feature strong frequency-dependent phenomena (Fig. 11, 12, and S10†). The Cole–Cole plots are shown in Fig. 11 and 12, and they were analyzed using the generalized Debye model.^16^ The obtained *α* values vary from 0.04 to 0.24 for 2a and 0.14 to 0.68 for 2b, indicating a rather broad distribution of relaxation times.

## Conclusions

A new functional nitronyl nitroxide, namely NIT-Ph-*p*-OCH_2_trz (1), and its Tb complexes (2) were successfully obtained. Complexes 2a and 2b displayed two different 1D chains. In particular, 2a showed a rare ladder-like arrangement of alternating 2p–3d–4f spins with exchange interactions propagating along the rails. Both 2a and 2b exhibit a slow magnetic relaxation behavior below 3.0 K. This study illustrates that functionalized nitronyl nitroxide radicals are promising building blocks for synthesizing 2p–3d–4f heterotrispin systems.

## Conflicts of interest

There are no conflicts to declare.

## Supplementary Material

RA-010-D0RA00018C-s001

RA-010-D0RA00018C-s002
