# Supplementary material for: A nitronyl nitroxide and its two 1D chain Cu–Tb complexes: synthesis, structures, and magnetic properties
Source: RSC Adv. 2020 Feb 27;10(14):8490–6. doi: 10.1039/d0ra00018c (PMC9050009; doi:10.1039/d0ra00018c)
Supplement: RA-010-D0RA00018C-s001 [file RA-010-D0RA00018C-s001.pdf]

Electronic Supplementary Material (ESI) for RSC Advances.
This journal is © The Royal Society of Chemistry 2020

A novel nitronyl nitroxide and its two 1D chain Cu-Tb complexes: synthesis, structures and magnetic properties

Meng Yang*, Xiaohong Liang, Yandie Zhang, Zhijian Ouyang, Wen Dong*

Guangzhou Key Laboratory for Environmentally Functional Materials and Technology, School of Chemistry and Chemical Engineering, Guangzhou University, 230 Wai Huan Xi Road, Guangzhou Higher Education Mega Center, Guangzhou 510006, P. R.China.

Table S1 Selected bond lengths [Å] and angles [°] for compound **1**.

Bond distances			
N(1)–O(1)	1.275(3)	N(1)–C(10)	1.354(3)
N(1)–C(11)	1.493(3)	N(2)–O(2)	1.290(2)
N(2)–C(10)	1.354(3)	N(2)–C(12)	1.500(3)
C(11)–C(13)	1.527(3)	C(11)–C(14)	1.523(3)
C(12)–C(15)	1.519(3)	C(12)–C(16)	1.534(3)
C(10)–C(1)	1.454(3)	C(4)–O(3)	1.380(3)
C(7)–O(3)	1.425(3)	C(7)–N(3)	1.433(3)
Angles			
O(1)-N(1)-C(10)	126.7(2)	O(2)-N(2)-C(10)	126.6(2)
N(1)-C(10)-N(2)	107.5(2)	C(10)-N(2)-C(12)	112.6(2)
C(10)-N(1)-C(11)	112.7(2)	C(11)-C(12)-C(15)	115.0(2)
C(11)-C(12)-C(16)	114.0(2)	C(12)-C(11)-C(13)	113.2(2)
C(12)-C(11)-C(14)	115.3(2)	C(3)-C(4)-O(3)	124.0(2)
C(4)-O(3)-C(7)	116.1 (2)	O(3)-C(7)-N(3)	107.1(2)
C(7)-N(3)-N(4)	120.4(2)	C(7)-N(3)-C(9)	129.8(2)
N(1)-C(11)-C(12)	101.2(2)	N(2)-C(12)-C(11)	100.3(2)
N(1)-C(11)-C(13)	106.1(2)	N(1)-C(11)-C(14)	109.4(2)
N(2)-C(12)-C(15)	110.0(2)	N(2)-C(12)-C(16)	106.4(2)

Table S2 Selected bond lengths (Å) and angles (°) for complex **2a.**

Bond distances			
Tb(1)–O(2)	2.345(3)	Tb(1)–O(4)	2.329(3)
Tb(1)–O(5)	2.354(4)	Tb(1)–O(6)	2.335(3)
Tb(1)–O(7)	2.361(4)	Tb(1)–O(8)	2.333(4)
Tb(1)–O(9)	2.340(3)	Tb(1)–N(5)	2.578(4)
Cu(1)–O(1)	2.409(4)	Cu(1)–O(10)	1.943(4)
Cu(1)–O(11)	1.952(3)	Cu(1)–O(12)	1.943(4)
Cu(1)–O(13)	1.951(3)	Cu(1)–N(4A)	2.467(4)
Angles			

O(2)-Tb(1)-O(4)	75.21(12)	O(2)-Tb(1)-O(5)	73.23(12)
O(2)-Tb(1)-O(6)	106.36(12)	O(2)-Tb(1)-O(7)	75.79(12)
O(2)-Tb(1)-O(8)	149.44(12)	O(2)-Tb(1)-O(9)	85.88(12)
O(2)-Tb(1)-N(5)	140.85(14)	N(5)-Tb(1)-O(4)	84.57(12)
N(5)-Tb(1)-O(5)	68.94(13)	N(5)-Tb(1)-O(6)	74.13(12)
N(5)-Tb(1)-O(7)	136.66(12)	N(5)-Tb(1)-O(8)	69.71(14)
N5(1)-Tb(1)-O(9)	119.77(13)	O(1)-Cu(1)-O(10)	94.38(16)
O(1)-Cu(1)-O(11)	88.46(14)	O(1)-Cu(1)-O(12)	88.31(16)
O(1)-Cu(1)-O(13)	91.22(14)	O(1)-Cu(1)-N(4A)	177.00(17)
N(4A)-Cu(1)-O(10)	88.31(16)	N(4A)-Cu(1)-O(11)	92.79(15)
N(4A)-Cu(1)-O(12)	89.03(16)	N(4A)-Cu(1)-O(13)	87.49(15)
Tb(1)-O(2)-N(2)	134.9(3)	Cu(1)-O(1)-N(1)	156.7(4)

A: x+1,y-1,z

Table S3 SHAPE analysis for Ln ions in complexes **2a** and **2b**.

Complex	Ln	SAPR-8	TDD-8	JBTPR-8	BTPR-8
**2a**	**Tb**	**0.863**	2.247	2.206	1.440
**2b**	**Tb1**	**0.999**	1.180	2.492	1.837
	**Tb2**	**0.989**	1.521	2.483	1.891
	**Tb3**	**0.597**	1.418	2.569	1.952

Table S4 SHAPE analysis for Cu ions in complex **2b**.

complex	VOC-5	TBTY-5	SPY-5	JTBPY-5
**Cu1**	0.985	4.327	**0.815**	7.194
**Cu2**	4.785	**2.254**	3.907	5.606
**Cu3**	2.788	3.128	**2.504**	6.214

Table S5 Selected bond lengths (Å) and angles (°) for complex **2b.**

Bond distances			
Tb(1)–O(1)	2.343(5)	Tb(1)–O(2)	2.373(7)
Tb(1)–O(3)	2.356(7)	Tb(1)–O(4)	2.344(9)
Tb(1)–O(5)	2.322(7)	Tb(1)–O(6)	2.355(8)
Tb(1)–O(7)	2.396(6)	Tb(1)–O(21A)	2.380(6)
Tb(2)–O(8)	2.371(6)	Tb(2)–O(14)	2.352(6)
Tb(2)–O(15)	2.370(7)	Tb(2)–O(16)	2.370(8)
Tb(2)–O(17)	2.340(7)	Tb(2)–O(18)	2.369(7)
Tb(2)–O(19)	2.340(8)	Tb(2)–O(20)	2.375(6)
Tb(3)–O(27)	2.373(6)	Tb(3)–O(28)	2.371(6)
Tb(3)–O(29)	2.359(7)	Tb(3)–O(30)	2.374(6)
Tb(3)–O(31)	2.352(6)	Tb(3)–O(32)	2.358(7)

Tb(3)–O(33)	2.353(7)	Tb(3)–O(34)	2.370(5)
Cu(1)–N(5)	1.979(7)	Cu(1)–O(10)	1.911(6)
Cu(1)–O(11)	1.957(6)	Cu(1)–O(12)	2.158(9)
Cu(1)–O(13)	1.939(6)	Cu(2)–N(10)	1.956(8)
Cu(2)–O(23)	1.908(8)	Cu(2)–O(24)	2.102(9)
Cu(2)–O(25)	2.027(7)	Cu(2)–O(26)	1.908(7)
Cu(3)–N(15)	1.968(7)	Cu(3)–O(36)	1.918(8)
Cu(3)–O(37)	1.954(7)	Cu(3)–O(38)	2.167(10)
Cu(3)–O(39)	1.926(7)		
Angles			
O(7)-Tb(1)-O(1)	85.4(2)	O(7)-Tb(1)-O(2)	138.9(2)
O(7)-Tb(1)-O(3)	102.4(2)	O(7)-Tb(1)-O(4)	69.9(3)
O(7)-Tb(1)-O(5)	74.8(2)	O(7)-Tb(1)-O(6)	78.3(2)
O(7)-Tb(1)-O(21A)	149.3(2)	O(21A)-Tb(1)-O(1)	104.1(2)
O(21A)-Tb(1)-O(2)	71.1(2)	O(21A)-Tb(1)-O(3)	86.3(2)
O(21A)-Tb(1)-O(4)	139.9(3)	O(21A)-Tb(1)-O(5)	80.5(2)
O(21A)-Tb(1)-O(6)	77.1(2)	O(8)-Tb(2)-O(14)	104.6(2)
O(8)-Tb(2)-O(15)	72.1(2)	O(8)-Tb(2)-O(16)	138.4(2)
O(8)-Tb(2)-O(17)	83.0(2)	O(8)-Tb(2)-O(18)	79.4(2)
O(8)-Tb(2)-O(19)	78.8(2)	O(8)-Tb(2)-O(20)	149.6(2)
O(20)-Tb(2)-O(14)	84.9(2)	O(20)-Tb(2)-O(15)	137.6(2)
O(20)-Tb(2)-O(16)	70.6(2)	O(20)-Tb(2)-O(17)	104.3(2)
O(20)-Tb(2)-O(18)	75.7(2)	O(20)-Tb(2)-O(19)	77.0(2)
O(27)-Tb(3)-O(28)	77.07(19)	O(27)-Tb(3)-O(29)	73.8(2)
O(27)-Tb(3)-O(30)	83.16(19)	O(27)-Tb(3)-O(31)	142.6(2)
O(27)-Tb(3)-O(32)	107.7(2)	O(27)-Tb(3)-O(33)	71.4(2)
O(27)-Tb(3)-O(34)	144.4(2)	O(34)-Tb(3)-O(28)	74.81(18)
O(34)-Tb(3)-O(29)	77.1(2)	O(34)-Tb(3)-O(30)	108.24(19)
O(34)-Tb(3)-O(31)	71.4(2)	O(34)-Tb(3)-O(32)	82.9(2)
O(34)-Tb(3)-O(33)	142.5(2)	N(5)-Cu(1)-O(10)	89.2(3)
N(5)-Cu(1)-O(11)	164.0(4)	N(5)-Cu(1)-O(12)	103.7(3)
N(5)-Cu(1)-O(13)	90.9(3)	N(10)-Cu(2)-O(23)	90.2(3)
N(10)-Cu(2)-O(24)	129.4(3)	N(10)-Cu(2)-O(25)	140.6(4)
N(10)-Cu(2)-O(26)	91.6(3)	N(15)-Cu(3)-O(36)	88.5(3)
N(15)-Cu(3)-O(37)	154.9(4)	N(15)-Cu(3)-O(38)	118.3(4)
N(15)-Cu(3)-O(39)	91.6(3)		

A: x,y+1,z

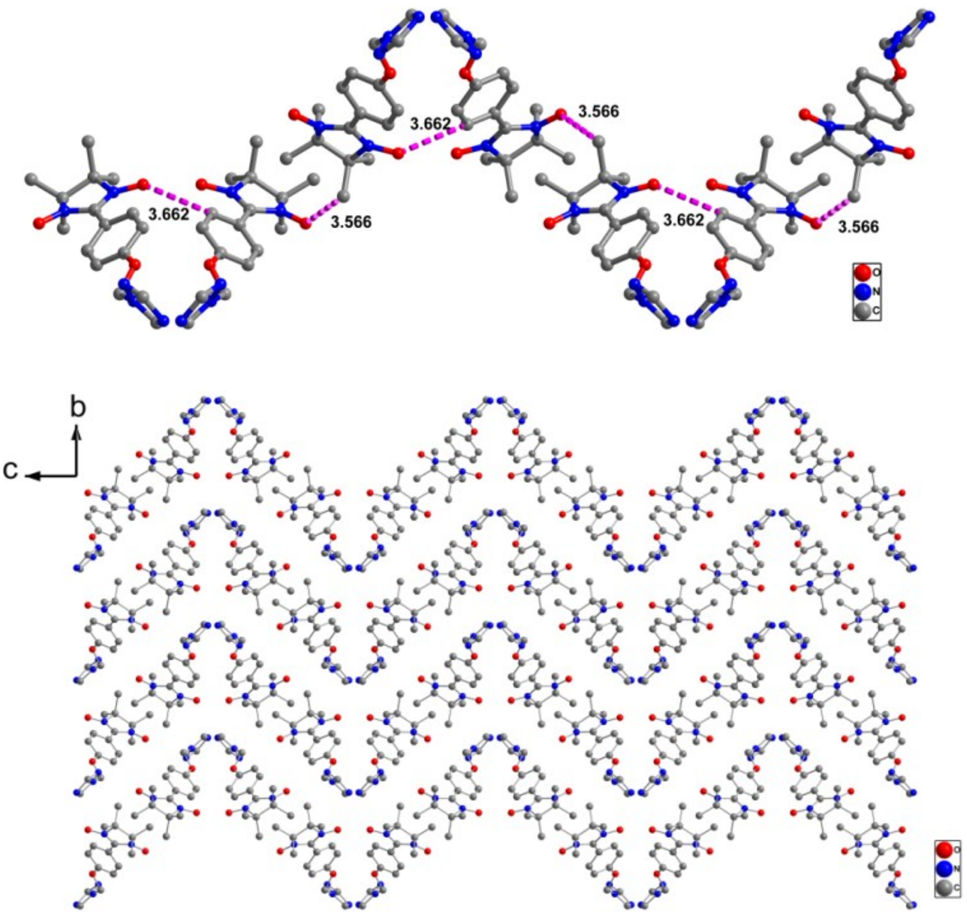

Figure S1. One-dimensional chain via hydrogen bonding and packing arrangement of **1**.

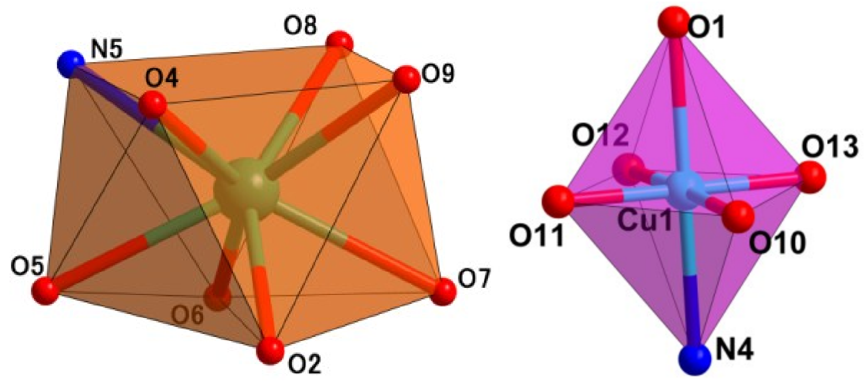

Figure S2 Local coordination geometry of Tb^{III} ion and Cu^{II} ion in complex **2a**.

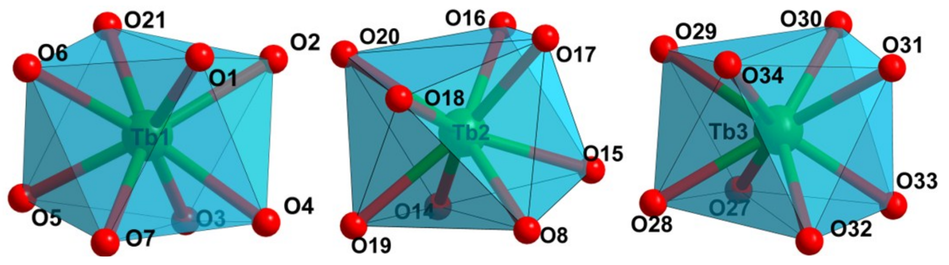

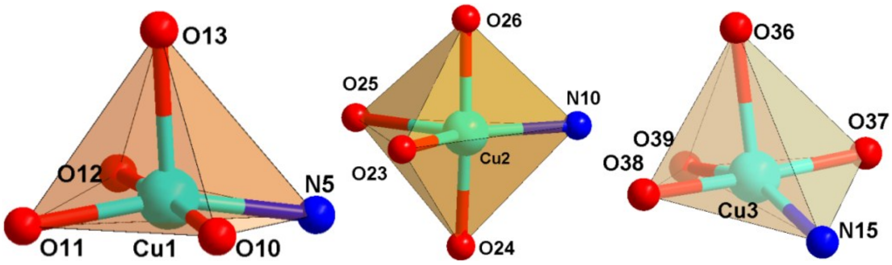

Figure S3 Local coordination geometry of Tb^{III} ions and Cu^{II} ions in complex **2b**.

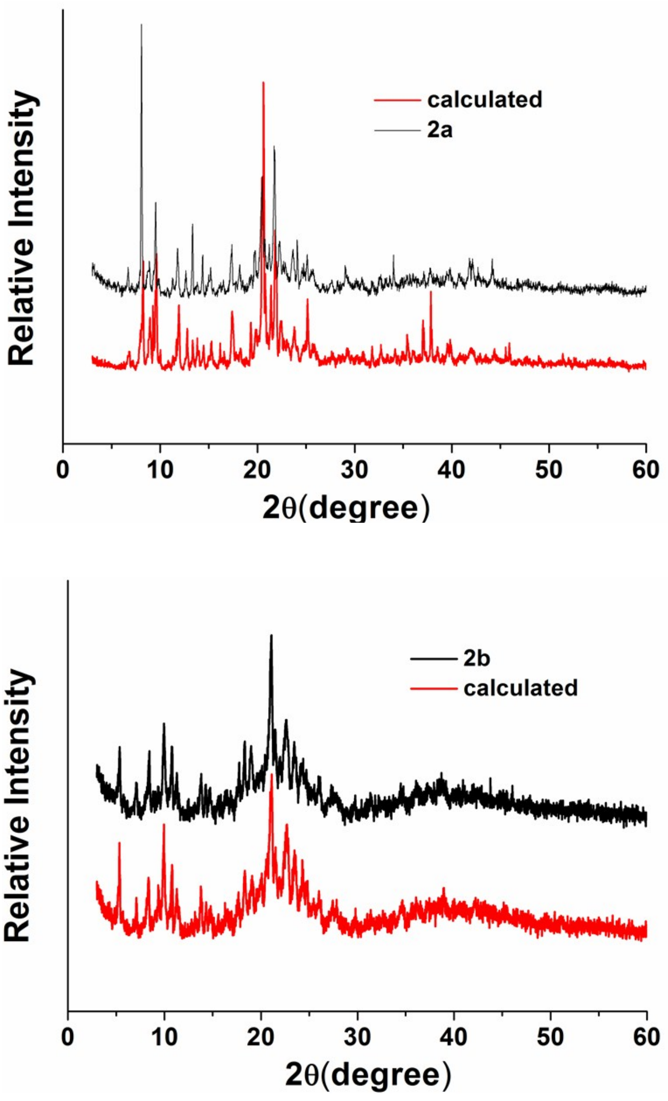

Figure S4 XRD patterns of complexes **2a** and **2b**.

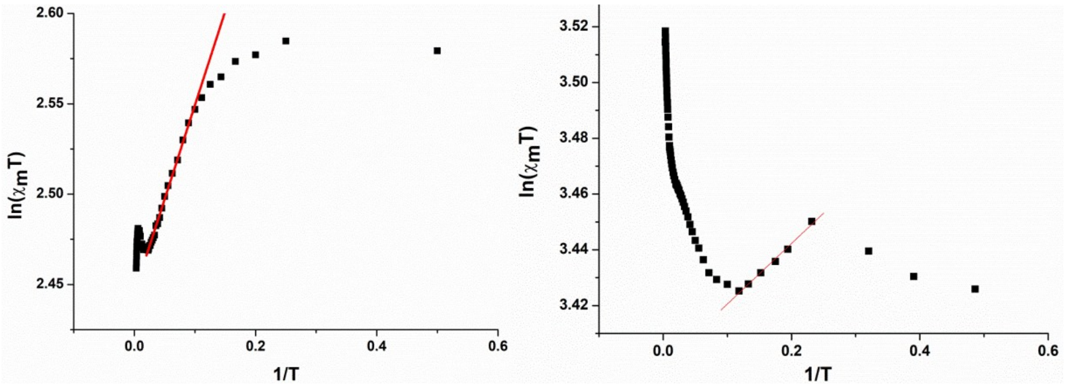

Figure S5 ln(χT) vs. 1/T plot for **2a** (left) and **2b** (right).

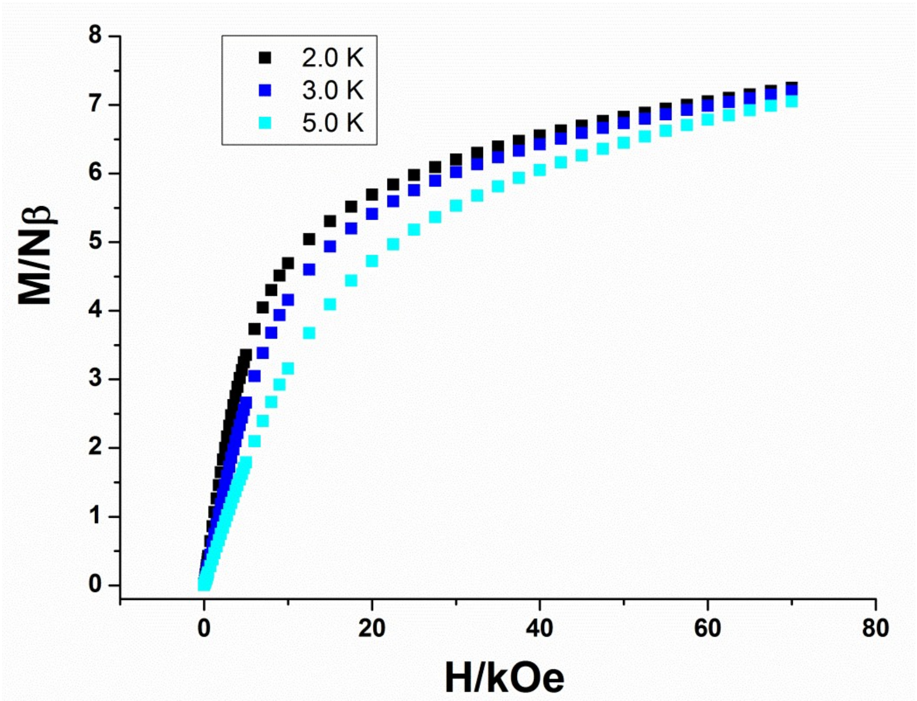

Figure S6 *M* versus *H* plot at 2, 3, 5 K for complex **2a**.

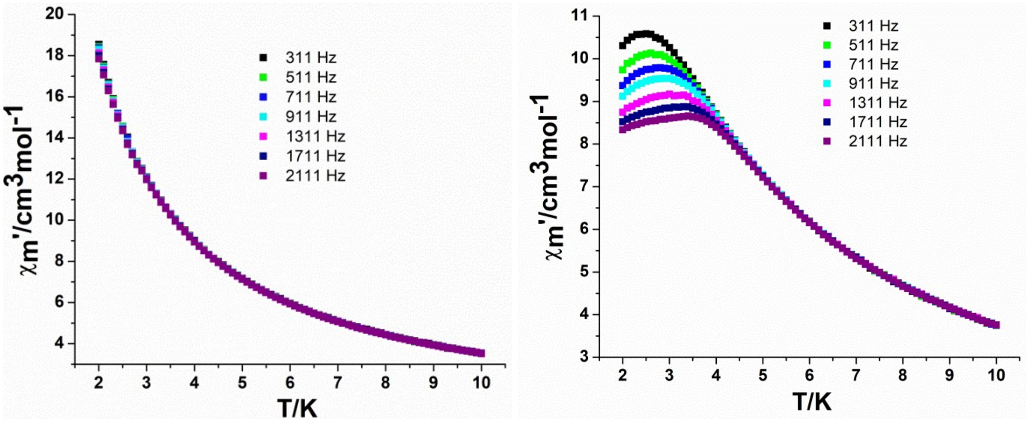

Figure S7 Temperature dependence of the out-phase in zero (left) and 2 kOe field (right) for complex **2a**.

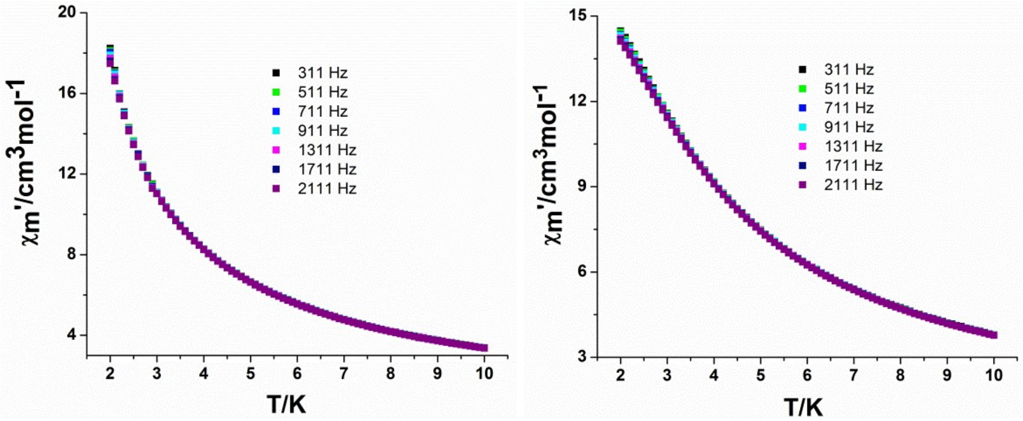

Figure S8 Temperature dependence of the in-phase in zero (left) and 2 kOe field (right) for complex **2b**.

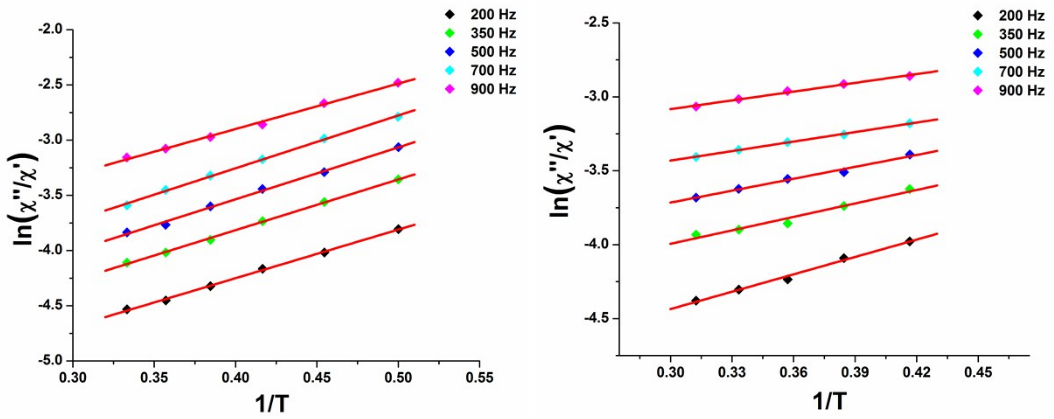

Figure S9 Natural logarithm of the ratio of χ''/χ' vs. 1/T for **2a** (left) and **2b** (right). The solid line represents the fitting results.

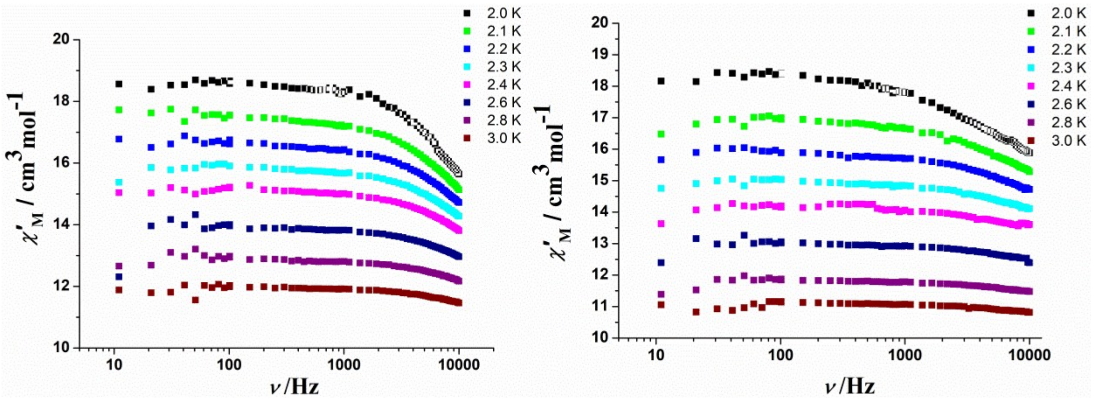

Figure S10 Frequency dependence of the out-of-phase components for the ac magnetic susceptibility in a zero field for **2a** (left) and **2b** (right).
